# Sensor Systems Based on FPGAs and Their Applications: A Survey

**DOI:** 10.3390/s120912235

**Published:** 2012-09-06

**Authors:** Antonio de la Piedra, An Braeken, Abdellah Touhafi

**Affiliations:** 1 Department of Electronics and Informatics (ETRO), Faculty of Engineering Sciences, Vrije Universiteit Brussel (VUB), Pleinlaan 2, Brussels 1050, Belgium; E-Mail: abdellah.touhafi@vub.ac.be; 2 Erasmushogeschool Brussel, Nijverheidskaai 170, Brussels 1070, Belgium; E-Mail: an.braeken@ehb.be

**Keywords:** wireless sensor networks, FPGA, 802.15.4

## Abstract

In this manuscript, we present a survey of designs and implementations of research sensor nodes that rely on FPGAs, either based upon standalone platforms or as a combination of microcontroller and FPGA. Several current challenges in sensor networks are distinguished and linked to the features of modern FPGAs. As it turns out, low-power optimized FPGAs are able to enhance the computation of several types of algorithms in terms of speed and power consumption in comparison to microcontrollers of commercial sensor nodes. We show that architectures based on the combination of microcontrollers and FPGA can play a key role in the future of sensor networks, in fields where processing capabilities such as strong cryptography, self-testing and data compression, among others, are paramount.

## 1. Introduction

In the last years, Wireless Sensor Networks (WSNs) have emerged in the academic environment, being transferred from military applications based on identification, classification and tracking of objects in the battlefield. Thanks to the flexibility of wireless links, WSNs are used in several fields [1] such as industrial control, precision agriculture, analysis of vital parameters in medicine, and dataset generation for modelling of ecosystems and prediction in environmental monitoring applications [2].

A sensor network consists of a set of sensor nodes made up of one or several processing elements, sensors, a small battery and a wireless transceiver which sends measurements from the sensors to a gateway that routes the data towards a logging system. Communications are typically based on the IEEE 802.15.4 standard [3] due to its focus on low-power communications and simplicity. However, 802.11 links are also used in gateways for high-speed data transmission. The upper layers are usually based on the ZigBee Alliance stack [4] or on optimized research layers such as S-MAC [5] or T-MAC [6] in the case of the Medium Access Layer (MAC).

However, sensor nodes suffer from several critical requirements: the fabrication cost of the node, energy consumption and its processing capabilities, both individual and distributed [7]. Energy efficiency in a sensor node is essential, given that either recharging or replacing the battery of hundred nodes can be a time-consuming task. Consequently, the main challenge in WSNs is reducing the power consumption of the nodes. A typical strategy consists of reducing the transmission of data among the sensor nodes [8]. Thus, an additional intelligent layer is required in order to provide an extra level of processing with the aim of compressing the sensor measurements into short messages, alerts or information about events. Other power minimization strategies consist of reducing the duty cycle, changing dynamically the frequency [9] and turning on the radio transceiver selectively [10]. In this respect, since the processing capabilities of sensor nodes are quite limited and rely on microcontrollers [11], Field-Programmable Gate Arrays (FPGAs) can play a key role.

Nowadays, FPGAs are crossing the 28 nm CMOS threshold (particularly those of Xilinx and Altera) and vendors provide optimized platforms in energy consumption that are able to run on ultra low-power such as the Actel IGLOO board. Such platforms can add extra processing capabilities to a typical sensor node with a reduced energy consumption if they are turned on when required by a microcontroller. Moreover, state-of-the-art FPGAs consist of powerful processing capabilities, based on ASIC arithmetic components such as high-speed multipliers and adders. Hence, the FPGAs can provide strong cryptography capabilities and high-speed acceleration of algorithms such as compression, image processing, and routing.

This survey explores the use and possibilities of FPGAs in sensor node architectures and their applications, focusing on the level of power consumption and the proper optimization of the current embedded resources of novel FPGAs.

In Section 2, we describe the current capabilities of FPGAs of the three main vendors, *i.e.*, Xilinx, Altera and Actel. Section 3 links the main challenges of sensor networks with the current capabilities of novel FPGAs and we propose several applications. Section 4 gives a survey of the state-of-the-art of research sensor nodes either based on FPGA, *i.e.*, standalone, or in combination with a commercial off-the-shelf (COTS) microcontroller. In Section 5, we summarize the features of the sensor nodes described in Section 4. Finally, we end in Section 6 with some conclusions.

## 2. Current Capabilities of Modern FPGAs

### 2.1. FPGAs vs. ASICs

FPGAs are made up of an interconnection of logic blocks in the form of a bidimensional array. The logical blocks consist of look-up tables (LUTs) constructed over simple memories that store boolean functions. Each LUT has a fixed number of inputs and is coupled to a multiplexor and a Flip-Flop in order to build sequential circuits. Likewise, several LUTs can be combined in order to implement complex functions.

The FPGA architecture makes it possible to implement any combinational and sequential circuit, which can range from a simple logic function to a high-end soft-processor [12]. In order to reduce the complexity of designing FPGA systems, Hardware Description Languages (HDL) such as VHDL and Verilog HDL are used. Likewise, the vendor's synthesizer seeks for an optimized arrangement of the FPGA's resources based on the hardware description (particularly the content of the LUTs and interconnection) during the process of mapping and routing in order to generate a bitstream, which is afterwards loaded in the platform.

In the past, FPGAs have been mainly used in signal processing and network packet analysis [13]. However, thanks to the high-speed embedded resources included in the FPGA such as DSP slices and fast memories, they are now also utilized for algorithm acceleration either as coprocessors or standalone systems, *i.e.*, System-On-Chips (SoCs). Moreover, several vendors reserve space in the FPGAs to custom soft hard-IP (HIPs) processors.

Besides the reprogramming capabilities, the usage of an FPGA as platform has several advantages in comparison to ASIC. Firstly, implementations can be updated in order to introduce a new feature, even partially and during run-time. Secondly, current FPGAs include high-speed multipliers and adders that work at the highest supported frequency. Furthermore, FPGAs track the Moore's law, *i.e.*, such platforms based on SRAM provide the advantages of the latest fabrication processes of ICs in terms of area reduction and energy consumption. Finally, their costs are much lower in comparison to ASIC fabrication of custom ICs. However, ASICs have much better performance and require much less area and energy. Thus, energy consumption is nowadays the main challenge of FPGAs vendors [14]. Likewise, vendors as Xilinx are substituting platform components by optimized ASIC HIPs in order to provide a better power balance [12].

### 2.2. Features and Platforms of FPGA Vendors

According to [15], the FPGA architecture has been changing from 2000, when such platforms were usually utilized for ASIC prototyping. As noted before, in the last years the main vendors have focused on reducing energy consumption using optimized HIPs. For instance, Xilinx and Altera based their platforms on a combination of coarse and fine grained components, that is, a combination of HIPs and small LUTs of 4 and 6 inputs.

Below, the features and platforms of the main vendors, *i.e.*, Xilinx, Altera and Actel, are briefly described.

#### 2.2.1. Xilinx

As Actel did in the past, Xilinx recently started to base their platforms on hard processor blocks. For instance, the Zynq-7000 platform [16] is based on a dual-core Cortex A9 processor that configures the FPGA itself, providing a SoC oriented platform focused on video processing and wireless communication. Likewise, Xilinx includes in its road-map the development of reconfigurable platforms for analog signal processing, similar to the mixed-signal FPGAs of Actel (Actel Smart Fusion).

Xilinx classifies their FPGAs in three series: Virtex (high-end), Artix (low-power), and Kintex (low-cost). The FPGAs are based on reduced energy consumption, 28 nm and of High-K Metal Gate (HKMG) fabrication process [17].

These heterogeneous platforms consist of fine and coarse grained components such as 6 input LUTs and powerful embedded blocks like 28 Gbps serial transceiver, 25 × 18 bits multipliers, 48-bit accumulators and pre-adders working at the maximum frequency allowed by the core. Moreover, Xilinx supports the 32-bit MicroBlaze soft-processors, based on the high-performance interconnection bus AXI.

On the other hand, several authors continue proposing designs using old-fashioned FPGAs from Xilinx such as the Spartan-3, Spartan-3E and Spartan-6 platforms due to its low-cost (particularly crucial in sensor nodes construction) and still powerful embedded blocks. Table 1 depicts the static power consumption of mid-range platforms of the different series of Xilinx. These values have been obtained via the Xilinx Power Estimator (XPE) tool.

#### 2.2.2. Altera

Altera shares the same road-map as Xilinx. It provides FPGAs based on 28 nm (V series [18]) technology SRAM and is aligned with the latest construction processes. Likewise, Altera provides 28 Gbps serial transceivers and 8 input LUTs. Moreover, the classification of Altera's FPGA is similar to Xilinx, which is Stratix (high-performance), Arria (mid-range) and Cyclone (low-cost). All the platforms are equipped with a variable amount of high-speed transceivers and DSP slices, similar to those of Xilinx. Moreover, Altera supports three soft-cores (Nios II, Cold Fire and ARM Cortex M1) in order to adapt their FPGAs to SoC-oriented platforms.

Table 2 depicts the static power consumption of the main FPGAs from Altera. However, since the number of different platforms is high, only the parameters from the last series (V) are depicted as well as those used by proposed designs in Section 4 (Altera Cyclone I and II, and Altera Stratix I and II). Those parameters have been estimated using the PowerPlay Early Power Estimators (EPE). Mid-range FPGAs have been chosen in order to compare their static power consumption with no loaded configuration as depicted in Table 1.

#### 2.2.3. Actel

Actel focuses on low-power and mixed-signal FPGAs. Their FPGAs are based on FLASH memory instead of SRAM. As a consequence, they have limited number of writings, are non-volatile and have a reduced power consumption, since the FPGAs are made up of less transistors and therefore their leakage-current is reduced in comparison to SRAM. Moreover, the proprietary Flash*Freeze technology allows to maintain their FPGAs (particularly IGLOO) in ultra low-power mode (*μ*W) without data loss. Therefore, the IGLOO platform fits completely in the sensor node architectures with on-board processing.

Furthermore, same as Xilinx and Altera, Actel supports the Cortex M1 soft-processor as well as the Cortex M3 as HIP. Actel's FPGAs are classified in three series: IGLOO (low-power), Pro ASIC (low-cost) and Smart Fusion (full-featured and mixed-signal focused) [19]. IGLOO is based on low-power, works between 1.2 V and 1.5 V and consumes between 2 *μ*W and 5 *μ*W in standby. This is extremely low compared to the two other series, ProASIC (3 mW) and SmartFusion (17 mW). Smart Fusion FPGAs are based on a HIP processor (Cortex M3) and signal processing blocks such as 12-bit ADCs/DACs, analog comparators and current monitors.

Table 3 depicts the static power consumption of the main FPGAs from Actel. It is worth noting that Actel provides two modes of turning off their platforms during execution, *i.e.*, Flash*Freeze and sleep, both reducing the power consumption enormously. The parameters from Table 3 have been estimated via the Actel Power Calculator tool.

### 2.3. Partial Reconfiguration

It is worth noting the dynamic reconfiguration capability of several FPGAs. Dynamic reconfiguration means modifying the system at run-time in order to change the content of the logic blocks as well as the interconnection configuration. This allows to reconfigure either portions or the whole content of the FPGA. Hence, the advantages of dynamic reconfiguration are twofold. First, it is possible to minimize the circuit area via the reconfiguration of the sequential parts of an algorithm, loading one step at a time. Second, it is possible to reduce the power consumption to the same extent as in the first case. However, not all vendors support the same type of reconfiguration. Moreover, reconfiguration is carried out according to different interfaces depending of the vendor. For instance, Xilinx Virtex FPGAs (4–7 series) support partial reconfiguration as well as the Artix-7 and Kintex-7 platforms. However Spartan and older Virtex series only allow to reconfigure sections of the FPGA based on differences of two bitstreams. Moreover, Xilinx allows to change the behaviour of several HIPs such as clock managers, Phase-locked Loops (PLLs), Analog to Digital Converters (ADCs) and transceivers using a set of memory-mapped registers.

Likewise, Altera's series V (including Stratix, Arria and Cyclone) support partial reconfiguration and dynamic reconfiguration of serial transceivers. On the other hand, since Actel platforms are not based on SRAM, partial reconfiguration is not supported. Nevertheless, Actel provides the reconfiguration of the FPGA's PLLs at run-time.

### 2.4. Summary

Hence, despite the gap between ASIC and FPGA in terms of energy consumption, the general vendor's road-map can lead very soon to a situation where sensor nodes benefit from the high-processing capabilities at low-cost. This will be achieved by either using low-power FPGAs with a lower computation capability (Actel) or by high-performance FPGAs with an accurate control to save power. In this respect, the mid-range Actel IGLOO platforms have a static power consumption between 0.030 mW and 0.012 mW at 1.2 V (Table 3). On the other hand, the Xilinx Artix-7 board has a similar static power consumption as the old-fashioned Spartan-3. However, it provides large space and better optimized blocks (Table 1). As can be seen from Table 2, static power consumption of the Altera V series is higher than those of the 7 series from Xilinx. Compared with the total active power consumption of commercial sensor nodes (Table 4), the Altera V series as well as the Virtex 4, 5 and 6 are ill-suited in sensor node construction since the static power consumption ranges from 0.206 to 1.977 W.

Finally, it is worth noting that, recently, Lattice Semi presented its ultra-low power FPGA iCE40, based on the Silicon Blue iCE65 FPGA and built on a 40 nm CMOS process. The logic elements of the FPGA are made up of a 4-input LUT and a FF The FPGAs are equipped with block RAM and one or two PLLs. There exists four models whose logic elements range from 640 to 7,680 and whose block RAM varies from 32 K to 128 K. However, as in the case of Actel IGLOO, they sacrificed specialized DSP at the cost of very small footprint and reduced power consumption.

The next section presents the current challenges in sensor networks and link them to the features of modern FPGAs. Those can be addressed in the future by constructing and initiating new paradigms in sensor network architectures, based on an extra layer of intelligence via high performance capabilities.

## 3. Challenges and Trends in Wireless Sensor Nodes

In 2003, Vieira *et al.* pointed out several limitations about the use of FPGAs in sensor node architectures [7]. First, they claimed that the power consumption of FPGAs made them ill-suited to be part of sensor nodes. Moreover, according to the authors, HDLs such as Verilog and VHDL cannot properly describe the complexity of a sensor node.

However, as described in Section 2, FPGAs have been thoroughly addressing the energy consumption problem in the recent years. That is especially the case for the IGLOO series from Actel that consumes 2 *μ*W in ultra low-power mode. Moreover, the vendor's synthesizers usually include optimized algorithms to save power such as intelligent clock gating in the case of Xilinx [20]. Also, the second presumed shortcoming, being the inability of HDLs, is not valid anymore. Due to the fact that FPGAs are nowadays SoC-oriented platforms, a soft-processor is able to communicate to custom blocks described in VHDL or Verilog and can easily implement communication stacks. Moreover, high-level synthesis tools, such as AutoESL and ROCC, can reduce the development time enormously. Another option is to base the sensor node architecture in a combination of microcontroller and FPGA.

In the end of this section, we describe eight current tendencies of sensor node technology and their corresponding challenges in WSNs. We also show how FPGAs can address them in respect to their communication, computation and reconfiguration capabilities. Table 5 depicts an overview of it.

### 3.1. Strong Cryptography for Sensor Nodes

According to [21], the security implementation in WSNs is one of the main key points. Standards, such as the IEEE 802.15.4 specification or the upper layers denned by the ZigBee Alliance, provide primitives for authentication, confidentiality and replay protection primitives. However, fixed implementations in forms of software or ASIC have two limitations. First, microcontrollers used in commercial nodes work at low frequency in order to reduce the energy consumption [22]. Therefore, in applications where a considerable amount of data must be authenticated and encrypted/decrypted, the microcontroller can be overloaded, thus depleting the battery. Second, given that vulnerabilities on security protocols and algorithms are discovered with a relatively high frequency, a software implementation (or ASIC) can become rapidly outdated. Recompiling and reprogramming hundreds of nodes in a WSNs can be a labor-intensive and a time-consuming task.

Hence, in this field, the use of FPGAs presents two main advantages:

1. The FPGA embedded resources, such as DSP slices and block RAM, can be used to accelerate arithmetic operations based, for instance, upon modular multiplications and reductions in the Galois Field (GF). That is the case of AES [23] and Elliptic Curve Cryptography (ECC) [24]. In this respect, several cryptographic accelerators have been proposed for sensor node architectures based on hyperchaos encryption [25], RC5 [26], involutional block ciphers [27] and implementations of the security suites of the IEEE 802.15.4 standard [28,29] among others. These implementations are described in Section 4.
2. Moreover, dynamic reconfiguration can play a key role into minimizing the obsolescence of cryptographic primitives, as proposed in [30]. Partial reconfiguration can be used in order to change, for instance, the curve parameters of ECC implementations in hardware.

### 3.2. Construction of Smart-Transducers

FPGA technology can be used in the implementation of smart transducers, either sensors or actuators. In so doing, sensors are provided with extra processing capabilities, according to the IEEE 1451 family of standards [31]. Thus, sensors are offered a particular interface, *i.e.*, Smart Transducers Interface Module (STIM), which provides ways for performing calibration, self-testing and configuration of the sensor according to different parameters. Therefore, smart transducers can group several sensors at once, sharing the same interface that can be accessed from a sensor node via the IEEE 1451.0 standard [31].

Likewise, several authors have already proposed the implementation of the IEEE 1451 standard on FPGAs in different measurement systems in order to provide extended capabilities to typical sensors [32,33].

In this respect, Rossi *et al.* implemented an IEEE 1451 protocol controller that serves as a sensor node and a smart sensor interface in order to provide advanced processing capabilities to a hydraulic demonstration system [34]. Furthermore, in order to support several types of analog sensors in a common acquisition board, [35] proposed the implementation of the IEEE 1451.4 [36] standard via the combination of FPGA and Field-Programmable Analog Array (FPAA).

However, partial reconfiguration has been rarely used on FPGA implementations of the IEEE 1451 family of standards and has been limited to proposals [37]. In this respect, an FPGA could be used in order to accelerate the complex techniques of self-testing and calibration of an array of different sensors, and it could be executed on demand via wireless communication, like WiFi, Bluetooth or ZigBee [38].

### 3.3. Reconfigurable Acquisition Boards

As [39] noted, a lot of sensors on the market are coupled with proprietary interfaces that can only be connected to specific data-loggers. Indeed, several sensor interfaces exist, both open and proprietary, and the lack of reconfigurability in sensor nodes and data-loggers undermines the use of a wide range of specialized sensors in a common platform [40].

However, commercial nodes exist which support digital buses such as SPI and I^2^C [22] via software. Nevertheless, substitution and connection of new sensors would require firmware recompiling and reprogramming. Hence, sensor substitution and maintaining would be an arduous task, given that some sensor networks consist of hundreds to thousands of nodes. In the case of FPGAs, using partial reconfiguration, several sensor interfaces can be supported and reconfigured remotely, in order to substitute a sensor without disrupting the node's connectivity. In this respect, Krasteva *et al.* [41,42] proposed the use of a low-cost FPGA (Spartan-3, XC3S200), in order to support several sensor interfaces such as PWM, I^2^C and ADC/DCA controllers. Such interfaces were implemented on a separate library with an interface based on the IEEE 1451 standard [31], which has rather limited partial reconfiguration support. The platform runs at 3 V power supply and consumes 60 mW in standby mode, 150 mW during reconfiguration and 240 mW in transmission. However, this implementation contains several shortcomings. The platform is limited in terms of reconfiguration since only difference-based partial reconfiguration is supported, where, through the comparison of two bitstreams, a third one is generated containing the differences of the columns among the two. Moreover, the FPGA reconfiguration is performed via JTAG, which is slower due to its serial nature. However, in [43], as the authors of the PowWow sensor node did (Section 3.5), the authors proposed an alternative processing board based on the MSP430 microcontroller and the Actel IGLOO FPGA, consequently sacrificing reconfiguration capabilities to attain a better power consumption.

To conclude, modern partial reconfiguration technology offers more possibilities in terms of programming area flexibility (Virtex 4–7). Due to the high cost of these platforms, this has not been exploited at the time of writing in order to support multiple interfaces.

### 3.4. Heterogeneous Sensor Networks: Supporting Several Physical Layers

As [44] noted, in environmental monitoring applications, sensors and data sources usually do not share the same nature of connectivity. For instance, the System for the Vigilance of the Amazon (SIVAN) [45] is based upon sensors of different types such as radars, environmental sensors and imaging systems. Therefore, such networks, composed of miscellaneous sensors and data sources, are generally called heterogeneous sensor networks [46]. These networks consist of high-speed links like imaging systems and low-speed measurements such as ground sensors. Thus, in order to reduce the network bandwidth and consequently reduce the energy consumption, nodes (particularly sensor gateways) are equipped with several network interfaces.

Providing a sensor gateway with a reconfigurable Software Defined Radio (SDR) physical layer makes it possible to switch, for instance, from 802.15.4 to 802.11 links, according to the required speed and parameters of the sensor network. That is the case of [47], where the authors proposed a 2.4 GHz receiver implemented in a low-cost Spartan-3 in order to demodulate 802.11 and 802.15.4 frames.

On the other hand, other works such as [48] have studied the support of several communication protocols in the same platform and the implementation of the RF network interfaces via commercial front-ends. In this respect, Kukkala *et al.* presented a WSN-to-WLAN bridge, implemented in an FPGA (Stratix II, EP2S60) and based on a multiprocessor SoC. This platform utilizes four Nios-II soft-processors to implement TDMA scheduling, MAC layer disassembling and assembling processes and the CRC-8 algorithm as well as custom logic in order to accelerate the AES encryption and CRC-32 algorithms.

At the time of writing, however, providing multiple communication standards via SDR and FPGA using partial reconfiguration in sensor networks remains an open problem.

### 3.5. IEEE 802.15.4 Physical Layer Implementations on FPGAs by Passive Power Conservation and Signal Processing

Pantazis *et al.* described how passive power conservation [49] could be applied on WSNs in order to reduce the power consumption of a sensor node, based upon the principle of turning the radio transceiver off when it is not transmitting [8]. This idea can be utilized in the implementation of the physical layer of the IEEE 802.15.4 standard, *i.e.*, Physical Layer Power Conservation (FLPC). Doing this on FPGAs has several advantages since independent units such as FIR structures, symbol mappers or synchronization modules can be turned off when not active. This technique, applied to other sensor node components with different granularity such as ADCs or sensors, is called Dynamic Power Management (DPM).

Moreover, Dynamic Voltage Scaling (DVS) can also be applied. Such technique is based upon changing the core's operation frequency and its voltage in order to reduce its energy consumption. Several implementations of DVS on FPGAs have been proposed [50,51].

In this respect, Berder *et al.* [9] proposed the design of a sensor node (PowWow) based on the MSP430 microcontroller and the Actel IGLOO FPGA (AGL125) where Dynamic Voltage and Frequency Scaling (DVFS) is employed. A ZigBee transceiver (CC2420) is directly coupled to the FPGA, which dynamically manages the transmission power according to the channel status and performs the packet processing tasks. If a valid frame is received, the FPGA wakes up the microcontroller. According to the authors, the main tasks are carried out by the FPGA since its power consumption is reduced in comparison to the MSP430. Finally, a DC/DC converter is used to perform Dynamic Voltage Scaling (DVS) on both the FPGA and the microcontroller.

Other power minimization technique consists of turning the radio transceiver off when the device is not processing incoming/outgoing packets. In this respect, Spinola *et al.* presented a wake-up receiver for WSN that wakes the transceiver up (based on FPGA) when a wake-up message is detected [10]. The wake-up receiver consumes 11 *μ*W.

### 3.6. Acceleration of Data Compression Techniques

According to [21], 80% of the energy consumed by a sensor node is due to data transmission. Hence, by minimizing the data size, a sensor node can save energy. As noted elsewhere, a typical strategy is to perform compression on the acquired data. Kimura *et al.* argued that the main problem of implementing compression algorithms on sensor nodes was the complexity and required time of execution [21]. Hence, FPGAs can be employed in this respect, for instance, to accelerate arithmetic applications via fast multipliers and adders [52].

Adaptive sampling techniques have also been proposed on WSNs in order to reduce the data size using prediction systems [53]. Hence, the coordinator node or gateway can create a model based on the last received measurements. This model is used to correlate new data with the aim of demanding the nodes to stop sending data or to reduce the frequency if values do not change [54]. Since a sensor network can consist of hundreds of nodes, the correlation algorithm can be accelerated via FPGA. In this respect, such adaptive sampling techniques could be applied to either subgroups or clusters in the networks in order to reduce the number of nodes required to be coupled with an FPGA, thus minimizing costs.

In [55], Debono *et al.* presented the implementation of a data prediction mechanism in order to reduce the data transmission in WSNs. Such technique is based on the least-mean-square (LMS) algorithm and was implemented on a Spartan-3E FPGA (XC3S100E). According to the authors, it was possible to reduce the communication with 90% of the time with an error of 0.5 degrees using a temperature sensor.

Since Wireless Multimedia Sensor Networks (WMSNs) are based on transmission of audio and video data, performing compression on the sensor nodes plays a key role [56]. These networks capture, process and transmit audio and video signals and operate on traffic enforcement, health care and ambient monitoring applications among others. In those fields, reducing the bandwidth is important since the size of data being transmitted is considerable. Thus, compression is either performed intraframe or interframe. In the latter, since predictive coding requires strong computation, FPGAs are used in order to implement hardware compressors based on MPEG, H.263, H.264 or even via distributed compression in the network [56].

We give in Section 4.2 an overview of existing implementations of FPGA-based sensor platforms in the context of multimedia.

### 3.7. Routing and Self-Location Algorithms Acceleration

Routing and self-location algorithms play a critical role in WSNs. When these algorithms are complex (e.g., large networks), they could be accelerated via FPGA. For instance, in [57], the authors proposed the implementation of the XMesh Routing Layer of Crossbow in a Xilinx CPLD. In this respect, Haider *et al.* implemented a Fuzzy Link Cost Processor (FLCP) in a Spartan-3 (XC3S5O) [58]. As a result, the authors extended a typical WSN gateway functionality in order to compute the link cost among two nodes via fuzzy logic. Once the gateway has computed the cost of all the possible links in the network, the routes can be estimated via the Shortest Path Algorithm. At 28 MHz, the system consumes 38 mW

### 3.8. WSNs Simulation

Simulation of WSNs for network load, physical layer transmission (e.g., bit error rate analysis) and channel modeling can be a complex task, given the number of nodes that can take part in a sensor network. In this respect, Nasreddine *et al.* [59] presented a WSN simulator implemented on a Stratix FPGA from Altera. Their implementation provides methods to model the sensor nodes and the utilized protocols as well as to manipulate the communication channel in order to estimate the bit error rate. Likewise, Zhang *et al.* [60] presented a simulator that allows to estimate the differences and advantages of executing an algorithm on a low-power microcontroller and in an FPGA. This system allows to define the behaviour of a flexible node (FPGA and microcontroller) that runs TinyOS. The simulator integrates SimulAVR and GEZEL. SimulAVR determines the microcontroller results. GEZEL is a HW/SW codesign environment for describing the FPGA implementation.

### 3.9. Summary

In this section, we have articulated the main challenges of sensor node construction and how FPGAs can address them. Table 5 depicts the requirements of each challenge in terms of communication, computation and reconfiguration. In this respect, an advised platform is also presented in each case. The Xilinx Artix-7 FPGA provides an optimal balance on reduced power consumption and reconfiguration capabilities and it also offers high speed transceivers and powerful DSP slices. Hence, this platform can be suitable for challenges 3.1–3.3 and 3.5.

On the other hand, the IGLOO platform can provide algorithm acceleration at ultra low-power, nevertheless lacking the capabilities of the Xilinx DSP slices.

Finally, the Virtex-7 platform can be also suitable or hardware construction in heterogeneous sensor networks, e.g., gateways, since it is equipped with several high-speed transceivers and has a large area.

## 4. A Survey on FPGA-Based Sensor Systems

In Section 3 we have presented the current challenges on WSNs that either start to be addressed at the time of writing or could be addressed using FPGAs. On the other hand, in this Section we describe the existing FPGA-based sensor nodes in most important application areas, *i.e.*, multimedia applications, industrial control, environmental monitoring and security. We first start with some general proof of concept designs.

### 4.1. Proof of Concept Designs

In this section, general designs of research sensor nodes are scrutinized. We describe the implementations and results of standalone platforms based on SoC from [61–64].

In [62], Wei *et al.* presented a sensor node based on SoC. Such SoC is based on the OpenRisc 1200 processor and executes the *μ*/OS II RTOS and Wishbone bus. The SoC consists of three main components: an acquisition unit that is fed from a digital thermometer coupled to the FPGA, the OpenRisc firmware that processes the temperature and a logic block that interfaces a ZigBee transceiver based on RS-232 (SHUNCOM SZ05-STD). The utilized FPGA is an Altera Cyclone II (EP2C70). The system consumes 221 mW We note that the power consumption during transmission and reception was not detailed. Nevertheless, given that the FPGA only interfaces several components (digital thermometer and ZigBee transmitter), it seems to be an overkill, since its inner embedded resources are not used and the same task can be performed using a low-power microcontroller. Also, [61] has the same problem. This sensor node is based on an Altera Cyclone I (EP1C6) that transmits the information via Bluetooth. Nevertheless, the authors did not provide information about the energy consumption of their platform. Moreover, Bluetooth is ill-suited in WSNs usage, since the IEEE 802.15.4 has a better approach for low-distance/low-power communications and has been adopted by the Zigbee Alliance for sensing applications.

The authors of [63,64] presented other standalone platform based on FPGA for a sensor node, where the Zefant XS3-2000 platform based on a Spartan-3 (C3S2000), CPLD and 128 Mb FLASH memory connected to a Xemics radio transceiver is used. However, information about the used CPLD is not provided. The implementation is based on the LEON2 32-bit processor. This platform was proposed in order to prototype smart sensor nodes with advanced processing capabilities and with extended debugging capabilities in the FPGA. Moreover, the FPGA allows to interface different types of sensors as well as to implement different MAC protocols. According to the authors, the platform consumes between 0.7 W and 1.1 W at 11 MHz, running the LEON2 SoC. A Reconfigurable Function Unit (RFU) was attached to the LEON2 system. The RFU consists of optimized modules to perform Galois Field (GF; arithmetic and inversion operations. The RFU is used from the processor through special implemented instructions.

The authors measured different implementations of algorithms in both software and custom logic. Implementations of CRC-8, AES key generation and encryption, and Broadcast Channel (BCH) decoding showed that custom logic reduce the energy consumption and execution time. Hence, AES encryption consumed 104.9 nJ (plus 43.1 nJ during the FPGA reconfiguration) *versus* 640.6 nJ in the processor. Furthermore, BCH decoding required 1,208.7 nJ in software in comparison to 245.5 *μ*J in hardware (plus 43.4 nJ during reconfiguration).

However, despite the reduction of power consumption by executing algorithms in the RFU instead of the processor, the advantages that provide a combination of low-power microcontroller and FPGA are twofold. First, the FPGA can be turned off when not needed in order to save power. Second, the FPGA can run independently at a different frequency than the microcontroller, which allows to turn it on during a shorter interval, searching for a balance between power consumption and speed. Consequently, the microcontroller can run at a lower frequency and the FPGA utilization can be optimized. Nevertheless, the reconfiguration time cannot be negligible in platforms based on a combination of microcontroller and FPGA, since external configuration of the FPGA requires more time [41] as well as data transmission between the FPGA and the microcontroller.

### 4.2. Multimedia Applications

This section comprises research sensor nodes for image compression ([65–69]), audio processing ([70,71]), image processing and task scheduling for multimedia applications via the FPGA ([71–75]).

Tanaka *et al.* [71] presented a hardware architecture of a sensor node based on an AVR ATMega microcontroller and an IGLOO FPGA (AGL600). The FPGA is utilized in order to perform a 512-points FFT The communication between the FPGA and the microcontroller is done at 4 MHz in parallel (8-bit). Power consumption of the platform in standby is 35.97 mW and 13.26 mW in sleep. The FFT execution on the FPGA consumes 12.36 mW, far less than the power consumption on the processor (382.94 mW).

In [65], the authors proposed Compressive Sampling (CS) in a Spartan-3 FPGA (XC3S400) in order to be used on WSNs. CS presents several advantages in comparison to traditional sampling methods, since it allows to reconstruct certain signals using a reduced number of samples. Recent works concerning the utilization of CS in WSNs appear promising in terms of energy efficiency [76,77]. However, the power consumption of the aforementioned design was not provided by the authors.

In [66], Kaddachi *et al.* presented the design of a sensor node with an FPGA coprocessor attached based on a Virtex-5 (XC5VLX0330T) that performs image compression via Loeffler DCT transform and controls an attached CMOS image sensor. At 100 MHz, the system consumes 364.85 mW. Moreover, the authors compared the power consumption with a similar implementation on a MICA2 sensor node, consuming 0.42 mJ *versus* 37.826 mJ in the commercial node.

Zhiyong *et al.* [67] presented a standalone sensor node based on an Altera Cyclone II FPGA (EP2C3) for image processing. The node is equipped with a 2.4 GHz NRF24L01 transceiver and a CMOS image sensor. The JPEG Baseline is implemented on the FPGA, in a SoC based on the Nios II soft-core. However, this implementation contains the same problems as those related in Section 4.1. JPEG Baseline could be better implemented in an IGLOO Board, consuming less power, and to be controlled by a low-power microcontroller coupled with a 802.15.4 transceiver.

Chefi *et al.* presented an FPGA-based coprocessor for image compression in sensor nodes based on the Haar Wavelet Transform (HWT). The implementation was done in a Virtex 5 FPGA, requiring 1.09 mJ for image encoding at 139.49 MHz and 267 mW.

In [70], the authors proposed a sensor node for optimized voice data transmission. The FPGA (Spartan-3, XC3S100E) implements the 802.15.4 MAC layer with a modification in the packet header, which gives priority to voice packets. An Atmel AT91 controls the audio sensor. However, the authors did not provide the energy consumption details of the platform.

Khursheed *et al.* [72] presented a sensor node for magnetic particles detection and classification in a liquid. The sensor node is based on the commercial platform SENTIO32 and an FPGA. The SENTNIO32 platform is a sensor node developed by the Mid Sweden University based on an AVR32 and a CC2520 802.15.4-based transceiver. The authors tested the low-cost Spartan-6 and the ultra low-power IGLOO FPGA. The FPGA performs background subtraction, object identification and extraction and TIFF compression. The FPGA processing consumes 5.93 mW (IGLOO). However, the SENTIO32 platform consumes 132 mW during transmission. Moreover, if all the image processing tasks are done in the microcontroller, it consumes 77.5 mW, 12 times more than on FPGA.

In [75], the authors presented a sensor node where the image processing is executed on a Spartan-3 FPGA (XC3S1000). The Running Gaussian Average algorithm is implemented in order to perform background subtraction and object extraction. The FPGA consumes 8.57 mW at 137.44 MHz.

Finally, Turcza *et al.* presented the design and implementation of an image processing core for a wireless capsule endoscopy (WCE) [69]. The core consists of a camera interface, an image compressor for color filter array (CFA) data and an error corrector encoder. The implementation has been done in a Silicon Blue iCE65 FPGA. The core, clocked at low frequency (24 MHz), consumes 9.6 mW at 1.2 V. The authors, in order to avoid complex image compressor calculations and maintain the power consumption low, have implemented an image compressor that operates directly on the data from the image sensor (CFA) based on an algorithm developed by Turcza *et al.* [78].

However, FPGAs are not only used in order to accelerate fixed tasks. Given that partial reconfiguration is supported in several FPGAs, some authors have proposed algorithms in order to optimize image processing in systems based on partial reconfiguration. In this respect, [73] presented a batch processing methodology for images to be used on sensor nodes with partial reconfiguration capabilities. The system is based on a strategy called ECfEE (Energy Efficient and Computation Efficient with Frequency Adaptation). The goal of the strategy is to find an efficient partition strategy to execute the maximum of tasks at a frequency that minimizes the overall power consumption. However, the authors did not provide the energy consumption of the platform application (edge detection and DES encryption).

In [74], a video sensor node equipped with energy harvesting is used to implement a task scheduler based on the available energy and past experiences in order to reconfigure the FPGA accordingly. The sensor node is based on an Atmel FPSLIC platform, which consists of an FPGA (AT40K) and 8-bit AVR microcontroller. However, given that the FPGA does not support partial reconfiguration, it must be completely reconfigured, consuming enough energy to deplete the battery rapidly. On the other hand, acceleration of tasks in hardware consumes 4.48 mJ (image thresholding) whereas it required 8.93 mJ in the AVR microcontroller. In this case, the FPGA reconfiguration requires 25 mJ. Moreover, performing edge detection, the AVR microcontroller consumes 28.08 mJ whereas the FPGA only consumes 6.60 mJ. Nevertheless, reconfiguring the FPGA with the edge detection algorithm requires 37.4 mJ.

To summarize, FPGAs, either low-power (e.g., IGLOO) or high-performance (Virtex-5), behave better in power consumption than processing on commercial nodes (MICA2) and 8-bit microcontrollers (AVR) [66,71]. In other cases, power consumption never exceeded 10 mW when performing image compression and processing on IGLOO platforms [72].

### 4.3. Industrial Control

Koskinen *et al.* proposed a sensor node based on an Actel FPGA and an 8-bit AVR microcontroller to be used in two WSNs for infrastructure measurement and analysis [79]. The first one consisted of a ground vibration monitoring application. The FPGA processes the signals and the AVR transfers the data and puts the FPGA into sleep mode when it is not required. The radio is based on 868 MHz and the sensor board is equipped with a 16-bit ADC and an analog signal processor. In order to detect ground motions, two MEMS accelerometers were used.

The second application is based on the wheel deformation measurement of trains via axial and radial forces estimation using strain gauges. The measurements are filtered on the FPGA, which consumes 60 mA during measurements and 10 *μ*A in standby.

### 4.4. Environmental Monitoring

This section describes sensor nodes based on object identification and tracking in the environment [80,81], pest control [82] and fire detection in woods [83].

In [80], the authors proposed situation-based reconfiguration in sensor nodes via FPGAs with partial reconfiguration capabilities (Virtex 4 FX100). They implemented Kalman filters in order to track environmental targets such as animals. Thus, each sensor must measure velocity and acceleration. According to the situation, depending on the type of object and the type of operation stage, several Kalman configurations exist. Therefore, the authors partially reconfigure the FPGA in order to reduce the power consumption. According to the authors, the base system has a power consumption of 1.794 W. The authors claimed that the power consumption is reduced about 5%–25% in comparison to a fixed implementation.

Liu *et al.* [81] presented a sensor node for bird call identification based on FPGA (Spartan 3E, XC3S1600E) that implements a MicroBlaze-based SoC. Such SoC implements a peripheral that performs bird call detection based on Linear Prediction Cepstral Coefficients (CPCC) in order to reduce the data dimension and extracts the features and Dynamic Time Warping (DTW) for performing template matching. The system, clocked at 50 MHz, consumes 2.85 W.

Jelicic *et al.* [82] presented the WSNs MasliNET, which is focused on pest detection in olive groves. Each sensor node is based on an Atmel Amp module consisting of an 8-bit AVR microcontroller and an IEEE 802.15.4 transceiver, connected to an FPGA Actel Pro ASIC 3 that deals with a CMOS sensor and perform image processing. The node consumes 87.12 mW in active mode (18.4 *μ*W in sleep mode) whereas the coordinator consumes 190 mA in active state (5 mA in sleep mode). The nodes require 3.3 V.

Finally, in [83], the authors presented the design and implementation of a sensor node for fire detection in woods. It is based on an Altera Cyclone II (EP2C70) FPGA that implements an SoC based on the Nios II processor, temperature sensor and Bluetooth transmitter PTR4500. However, this system presents a similar problem as [61] (Section 4.1). The FPGA is an overkill since its processing capabilities are not scrutinized and the Bluetooth technology is not appropriate for WSNs.

### 4.5. Safety and Security Applications for Sensor Nodes

This section is composed of sensor nodes focusing on safety ([84–86]) and security implementations for sensor node architectures ([25–27,30,87,88]).

In [84], Zhang presented the design of a sensor node based on an FPGA Altera Cyclone II (EP2C5) in order to be used in surveillance of a fabrication zone of aircrafts. The node is composed of an NRF2401 transceiver (2.4 GHz) and the system is based on a Nios-II soft-core. However, the author did not provide energy consumption results.

Latha *et al.* presented a WSN [85] focusing on surveillance and motion detection based on two levels of nodes. First level nodes consist of a Microchip microcontroller coupled with proximity and ultrasonic sensors. The microcontroller sends the data via 433 MHz ASK radio link to the second layer of nodes. These nodes comprise a Cyclone II (EP2C3) FPGA which performs motion detection, edge detection and image compression. The authors did not provide energy consumption results.

In [86], the authors presented the implementation of a people counter system accelerated on an IGLOO FPGA for WSN applications. The FPGA performs a Dijkstra-based algorithm at 10 MHz and consumes 5 mW.

Several cryptographic accelerators have been proposed for sensor nodes based on FPGAs whose applications range from acceleration based on embedded resources to partial reconfigured ones.

Elliptic Curve Cryptography is usually proposed as key agreement protocol in WSNs since it utilizes smaller keys, is faster and consumes less energy in comparison with other public key cryptosystems. In this respect, Peter *et al.* [30] presented a cryptographic accelerator for Elliptic Curve Cryptography (ECC) that supports various types of NIST curves via partial reconfiguration using the sensor node presented in Section 4. Moreover, Al-Somani *et al.* proposed the implementation of an ECC core for binary fields on an IGLOO FPGA (AGLN250V2) for a sensor node clocked at 27 MHz [89]. Besides that, Houssain *et al.* presented a survey of hardware implementations, ASIC and FPGA, focusing on WSN [87]. Also, Hamalainen *et al.* reviewed the state-of-the-art on AES designs focusing on sensor nodes [90].

The IEEE 802.15.4 standard [3] provides cryptographic primitives in the MAC layer in order to ensure confidentiality, authentication and replay protection. Likewise, several variations of AES [91] in counter-mode (CCM) can be utilized in ascending levels of security [92]. In this respect, several authors have proposed FPGA implementations of the IEEE 802.15.4 security suite. Hamalainen *et al.* presented a compact AES architecture in an Altera Cyclone I (EP1C4F) [28]. Clocked at 50 MHz, the core consumes 98.92 mW and can process up to 57 Mbps. The authors compared the features of the core with a software implementation of AES in an ARM966E-S microprocessor which consumes 140 mW and processes 16 Mbps, clocked at 200 MHz.

Likewise, Song *et al.* [29] presented an accelerator for 802.15.4-based sensor nodes. It consists of a compact AES core coupled with a Content Address Memory (CAM) which stores the keys and addresses the nodes in the WSN. It was implemented in an Altera Stratix I (EP1S10F), clocked at 3 MHz. It has a throughput of 600 kbps and consumes 29 mW.

Finally other cryptographic primitives have been proposed for sensor nodes, such as hyperchaos encryption (Spartan 3E, XC3S500E) [25], Skipjack and involutional block ciphers [27,88].

Zhang *et al.* proposed to implement two involutional block ciphers (particularly KHAZAD and BSPN) in a low-cost Spartan-3 FPGA (XC3S200) [27]. Given that they are involutional, that is, using the same algorithm for encryption and decryption, the authors claimed that they are suited to be implemented in area-constrained systems. The implementations have been compared with implementations in a low-cost microcontroller, an 8-bit ATMega 128. Likewise, the BSPN algorithm consumes 20.45 mW (5.12 nJ) and KHAZAD 32.39 mW (9.60 nJ) in the FPGA *versus* 1,373 nJ in the ATMega per block.

Finally, Eryumaz *et al.* [88] proposed the implementation of the Skipjack encryption algorithm for WSNs on a Spartan-3E (XC3S500E) FPGA clocked at 20 MHz. The power consumption of the implementation in the FPGA required 98.9 mW and 0.32 *μ*J per block. The authors compared their implementation with another implementation on a Microchip 16F84A microcontroller which consumes 82 mW and required 44.45 *μ*J per block.

## 5. Summary

In this section, the power consumption results of the sensor node architectures described in Sections 3 and 4 are reviewed and compared.

We first describe the properties of some commercial sensor nodes. Then we distinguish between FPGA standalone sensor nodes, sensor nodes based on microcontroller and FPGA and FPGA accelerators.

### 5.1. Commercial Sensor Nodes

Table 4 depicts the construction details and total active power of the most important commercial sensor nodes, *i.e.*, motes. This will allow a better understanding of the improvement and impact of using FPGA-based nodes or sensor nodes coupled with FPGA coprocessors. The parameters from Table 4 are based on [11].

Typical platforms of commercial nodes are usually based on 8-bit (particularly Mica, Mica2Dot and Mica2) and 16-bit microcontrollers (MSP430) running at low frequency, with simple peripherals such as ADCs in order to connect external sensors, a small battery and a 802.15.4/ZigBee transceiver. As depicted in Table 4, the total active power ranges from 27 mW to 89 mW.

### 5.2. FPGA Standalone Sensor Nodes

Table 6 summarizes the research sensor nodes that were completely based on FPGA, *i.e.*, standalone FPGA platforms which are SoC-oriented.

According to Table 6, there exists a key problem. Most of the authors did not provide detailed information about the power consumption of the proposed platforms. Even worst, the operational frequency was rarely provided. Moreover, there are certain issues that can be pointed out. First, in comparison to Table 4, documents [62–64,81] have a very high power consumption (221 mW, 2,850 mW and 700 mW respectively). In those cases, the FPGAs can deplete the battery in very low time without the possibility of switching the board in sleep mode when it is not working. Moreover, their power consumption is up to 32 times higher than the MICA2 node (Table 4). Nevertheless, it would be interesting to estimate the energy reduction and optimization in SoCs based on Nios-II and MicroBlaze using advanced clock gating and other techniques, in order to turn off the system custom logic.

For obtaining an estimation of the power consumption of the proposed standalone sensor nodes, we have investigated the static power consumption of the platforms via the XPE and EPE tools with no configuration. Hence, the quiescent power consumption of [25,61,67,83,84] was 60 mW, 29 mW, 193 mW, 29 mW and 78 mW respectively. Consequently, these implementations on FPGA are all ill-suited for sensor node construction.

To summarize, it is worth noting that using an FPGA to do simple processing of sensors and communication with an external transceiver is an overkill ([61,62,83])—even more if the FPGAs are those of general use instead of ultra low-power, e.g., Actel IGLOO. If not, a microcontroller is generally enough to interface sensors and external transceivers, as the commercial sensor nodes depicted in Table 4.

Finally, authors of [61,83] utilized Bluetooth whereas 802.15.4/ZigBee is being adopted as networking standard in current WSNs due to its low-power approach.

### 5.3. Sensor Nodes Based on Microcontroller and FPGA

Table 7 depicts the details of sensor nodes based on a combination of microcontroller and FPGA in the form of a coprocessor. In order to estimate the advantages of using hardware acceleration instead of a software implementation based upon microcontroller, the improvement is provided when it was investigated by the authors.

According to Table 7, the best results are produced using the IGLOO FPGA, *i.e.*, [71,72], where the power reduction implementing an FFT and image processing tasks was 30 and 13 times smaller respectively. Moreover, the authors of the PowWow sensor node measured the power consumption of the CRC32 algorithm in both microcontroller and FPGA [9]. In the case of the MSP430, it required 3 *μ*J (20 mW) running at 8 MHz. On the other hand, the IGLOO board required only 0.004 *μ*J (5mW) clocked at 20 MHz. In the other cases, the controlled execution of FPGA implemented tasks was also acceptable. Hence, based on the FPSLIC platform, [74] performs video processing that requires 4.48 mJ in the FPGA, which is almost half compared with the microcontroller execution (8.93 mJ). However, as the authors claimed, the FPGA reconfiguration (which must be full since partial reconfiguration is not supported therein) consumes more power (25 mJ). Finally, [27] greatly improved the power consumption for the implementation of an involutional block cipher (particularly KHAZAD), consuming 8.60 nJ per block in comparison to the software execution (1,373 nJ per block). However, since the authors did not provide the execution time per block, the power consumption is only provided in nJ instead of mW.

As noted before, there are still designs using low-cost FPGAs such as the Spartan-3 or ProASIC 3 which could have been replaced by the IGLOO board if, as the respective manuscripts suggest, their embedded resources are not utilized.

Nevertheless, the power consumption of [41,42,71] sensor platforms are within the limits of commercial nodes, as depicted in Table 4. This shows that it is possible to construct an efficient sensor node via a combination of microcontroller and FPGA.

### 5.4. FPGA Coprocessors

Table 8 depicts the results of the FPGA coprocessors described in Section 4, which were proposed as part of the sensor nodes. As noted before, the power consumption on the IGLOO predominates. Besides that, even low-cost FPGAs (Spartan-3, XC3S1000) had a decent power consumption ([75]) at high frequency (137.44 MHz) performing the Running Gaussian Average in order to do background subtraction of a captured image. However, the consumption of the implementation is not coherent with the static power consumption of the platform, *i.e.*, 98 mW, according to XPE.

Finally, two high-end (Virtex) FPGAs were used. In [66] a Virtex-5 is utilized in order to implement the Loeffler DCT running at 100 MHz. Despite the high operating frequency, according to the authors, the implementation behaves better than the same implementation in the MICA2 nodes (0.42 mJ in comparison to 37.826 mJ in the MICA2 platform). Moreover, the use of a high-end FPGA in a sensor node can be justified since partial reconfiguration is usually available in such FPGAs. In this respect, document [80] performs Partial Reconfiguration over different configurations of Kalman filters which, according to the authors, produced between 5% and 25% power reduction.

Given the results of the two 802.15.4 cryptographic accelerators, it is possible to make a trade-off between throughput and energy consumption. In [28], the core is clocked at 50 MHz and processes 57 Mbps. However, its power consumption exceeds the total active power of all commercial nodes depicted in Table 4. On the other hand, document [29] minimized the power consumption reducing the clock at 3 MHz and providing a throughput of 600 kbps (2.4 times the ZigBee bitrate of 250 kbps). The core consumes 29 mW, compared to 38.52 mW for the previous design.

To conclude, since most of the authors (Tables 6–8) did not provide enough power consumption details, it is still difficult to make a thorough comparison between such experimental nodes and commercial ones.

## 6. Conclusions

In this manuscript, we have shown how novel FPGAs can fill the gap between the shortcomings in the commercial nodes and the research nodes in terms of extended processing capabilities.

The novel FPGAs started some years ago to cross the 28 nm threshold (Xilinx, Altera) and there even exist FPGAs completely optimized on low-power, such as the Actel IGLOO platform. In the case of Xilinx and Altera, the Arria and Artix boards look promising since both are coupled with powerful specialized blocks and have a static power consumption between 41 mW and 197 mW. However, for applications with less advanced calculus at high-speed, the IGLOO platform provides a limited static power consumption within the *μ*W and the mW range.

We have compared three groups of sensor nodes: standalone, combinations of microcontrollers and FPGAs and FPGA coprocessors for experimental nodes. However, those standalone architectures consumed too much in comparison with commercial sensor nodes. Moreover, they did not employ low-power FPGAs nor utilize the FPGA embedded resources. In the second case, several works showed that the execution of algorithms was faster on the FPGA in comparison to the microcontroller, and the power consumption was also reduced. Moreover, these works were mainly based on low-power FPGAs (Actel IGLOO) and low-cost ones. Finally, we have reviewed a group of FPGA coprocessors for experimental sensor nodes, whose power consumption analysis was within the power consumption of commercial sensor nodes and, in some cases, was several times reduced.

To summarize, the architecture of sensor nodes made up of low-power microcontrollers and FPGA coprocessor is promising, even applied to the challenges that were described such as the support for multiple interfaces and strong cryptography implementation.

Finally, in the future we will see if the partial reconfiguration property of an FPGA can play a key role on the construction of sensor nodes, if the costs and power consumption are reduced. Moreover, more analysis in terms of power consumption and cost must be done on research nodes, in order to effectively compare their capabilities and possibilities with the commercial ones.

## Figures and Tables

**Table 1.** Power consumption and operating voltage of Xilinx FPGAs.

**Platform**	**Model**	**Core Voltage (V)**	**Number of LUTs**	**Block RAM (KB)**	**Static Power Consumption (mW)**
Spartan-3	XC3S200	1.2	4,320	216	41
Spartan-3 E	XC3S250E	1.2	5,508	216	51
Spartan-6	XC6SLX100	1.2	101,261	4,824	67
Virtex-4	XC4VLX200	1.2	200,448	6,048	1,278
Virtex-5	XC5VLX220	1	138,240	6,912	1,985
Virtex-6	XC6VLX240T	1	241,152	14, 976	1,977
Virtex-7	XC7VX330T	1	326,400	27, 000	141
Kintex-7	XC7K160T	1	162,240	11,700	74
Artix-7	XC7A100T	1	101,440	4,860	41

**Table 2.** Power consumption and operating voltage of Altera FPGAs.

**Platform**	**Model**	**Core Voltage (V)**	**Number of LUTs**	**Block RAM (KB)**	**Static Power Consumption (mW)**
Cyclone	EP1C6	1.5	5,980	92	60
Cyclone II	EP2C8	1.5	8,256	165	40
Cyclone V	5CEFA9	1.10	301,000	12,200	206
Stratix	EP1S25	1.5	25,660	635	450
Stratix II	EP230	1.5	33,880	663	86
Startix V	5SEE9	1	840,000	12,800	880
Arria V	57GXMA1D	1	75,000	8,000	197

**Table 3.** Power consumption and operating voltage of Actel FPGAs.

**Platform**	**Model**	**Core Voltage (V)**	**Number of FFs**	**Block RAM (KB)**	**Static Power Consumption (mW)**		

					***Static***	Flash*Freeze	***Sleep***
IGLOO	AGL060V5	1.5	1,536	18	0.030	0.018	0.005
IGLOO	AGL060V2	1.2	1,536	18	0.012	0.012	0.004
ProASIC3	A3P060	1.5	1,536	18	3	–	0.10
SmartFusion	A2F500	1.5	11,520	24	17.43	–	–

**Table 4.** Details of commercial sensor nodes.

**Mote**	**Platform**	**Platform Details**	**Communication**	**Total Active Power (mW)**
Mica	Microcontroller	ATMegal03 @ 4 MHz	TR1000, ASK	27
Mica2Dot	Microcontroller	ATMegal28 @ 7.4 MHz	CC1000, FSK	44
Mica2	Microcontroller	ATMegal28 @ 7.4 MHz	CC1000, FSK	89
Tmote Sky	Microcontroller	TiMSP430 @ 8 MHz	CC2420, OQPSK	32
Telos B	Microcontroller	TiMSP430 @ 8 MHz	CC2420, OQPSK	32
Imote2	Microprocessor	Intel PXA271 @ 13–100 MHz	CC2420, OQPSK	86.8

**Table 5.** Challenges on WSNs.

**Challenge** / **FPGA feature**	**Communication requirements**	**Computation requirements**	**Reconfiguration requirements**	**Advised platform**
Strong cryptography (3.1)	-	The DSP slices of FPGAs can be used for high-speed execution, e.g., modular adders and inverters for GF computations.	In order to support different parameters and avoid obsolescence due to vulnerabilities and support different degrees of security.	Artix-7
Construction of smart transducers (3.2)	High-speed transceivers and mixed signal/analog blocks can be useful for implementing wireless interfaces of the sensors.	Custom arithmetic logic implementations on FPGA and DSP slices can accelerate calibration and self-testing algorithms.	Implementing calibration and self-testing when several sensors of different nature are connected to the same platform can save area.	Artix-7
Multi-interface acquisition boards (3.3)	-	-	In order to support multiple digital protocols and avoid recompilation if case of substitution or failure, e.g., SPI, I^2^C *etc.*	Artix-7
Heterogeneous sensor networks PHYs (3.4)	High-speed transceivers and mixed-signal and analog processors for baseband processors construction.	-	To support several DSP and communication blocks for different standards of communication.	Artix-7 / Virtex-7
Passive power conservation on 802.15.4 implementations (3.5)	-	-	Different blocks of transmission and reception chain can be optimized and loaded when required.	Artix-7
Data compression (3.6)	-	DSP slices for high-speed execution of algorithms.	-	Artix-7 / IGLOO (for ultra low-power)
Acceleration of routing and self-location algorithms (3.7)	-	DSP slices for high-speed execution of algorithms.	-	Artix-7 / IGLOO (for ultra low-power)
WSN simulation (3.8)	-	DSP slices for high-speed execution of algorithms.	-	Artix-7 / IGLOO (for ultra low-power)

**Table 6.** FPGA Standalone sensor nodes.

**Work**	**Platform**	**Model**	**Communication**	**Power consumption (mW)**	**Frequency (MHz)**	**Sensors**	**Application**
[61]	Cyclone I (Nios-II)	EP1C6	Bluetooth (RS-232)	-	-	-	General
[62]	Cyclone II (OpenRisc)	EP2C70	ZigBee (RS-232)	221	-	Temperature	General
[63,64]	Spartan-3	XC3S2000	ZigBee (XEMICS)	700- 1,100	11	-	General
[67]	Cyclone II (Nios-II)	EP2C3	nRF24 2.4 GHz ISM	-	-	CMOS image sensor	Multimedia
[81]	Spartan-3E	XC3S1600E	-	2,850	50	-	Environmental monitoring
[83]	Cyclone II (Nios-II)	EP2C70	Bluetooth (RS-232)	-	-	Temperature	Environmental monitoring
[84]	Cyclone II (Nios-II)	EP2C5	nRF24 2.4 GHz ISM	-	-	CMOS image sensor	Security
[25]	Spartan-3E	XC3S500E	ZigBee (CC2430)	-	-	-	Security

**Table 7.** Sensor nodes based on a combination of microcontroller and FPGA coprocessor.

**Work**	**Microcontroller**	**FPGA Platform**	**FPGA Model**	**Communication**	**Standby Power Consumption (mW)**	**FPGA Execution Power Consumption**	**Microcontroller Execution Power Consumption**	**Application**
[41,42]	ADUC841	Spartan-3	XC3S200	ZigBee (ETRX2)	60	-	-	General
[71]	ATMega	IGLOO	AGL600	-	35.97	12.36 mW	382.94 mW	Multimedia/FFT
[70]	AT91	Spartan-3	XC3S100E	-	-	-	-	Multimedia/Image Processing
[72]	SENTIO32 (AVR32)	IGLOO		ZigBee (CC2520)	-	5.93 mW	77.5 mW	Multimedia/Image Processing
[74]	8-bit AVR	FPSLIC	AT40K	-	-	4.48 mJ	8.93 mJ	Multimedia/Image processing
[79]	8-bit AVR	Actel	-	ZigBee	-	-	-	Industrial
[82]	8-bit AVR	ProASIC3	-	ZigBee	87.12	-	-	Environmental/Image Processing
[27]	8-bit AVR	Spartan-3	XC3S200	-	-	9.60 nJ	1373 nJ	Security/Cryptography
[9]	MSP430	IGLOO	AGL125	ZigBee (CC2420)	-	5mW	20 mW	Communication/CRC32

**Table 8.** FPGA coprocessors for research sensor nodes.

**Work**	**Platform**	**Model**	**Frequency (MHz)**	**Power Consumption (mW)**	**Application**
[58]	Spartan-3	XC3S5O	28	38	General / Routing Acceleration
[66]	Virtex-5	XC5VLX330T	100	364.85	Multimedia / Loeffler DCT
[75]	Spartan-3	XC3S1000	137.44	8.57	Multimedia / Running Gaussian Average
[65]	Spartan-3	XC3S400	-	-	Multimedia / Image Compression
[68]	Virtex-5	-	139.49	267	Multimedia / Image Compression
[80]	Virtex-4	FX100	209	1,794	Environmental Monitoring / Kalman Filters
[86]	IGLOO	-	10	5	Security / Dijkstra Algorithm
[28]	Cyclone I	EP1C4F	50	98.92	Security / AES
[29]	Stratix I	EP1S10F	3	29	Security / AES
[88]	Spartan-3E	XC3S500E	20	98.9	Security / Skipjack
[89]	IGLOO	AGLN250V2	27	-	Security / ECC
